# Frailty as a predictor of mortality in older adults within 5 years of psychiatric admission

**DOI:** 10.1002/gps.5278

**Published:** 2020-02-23

**Authors:** Carolien E. M. Benraad, Miriam L. Haaksma, Mieke H. J. Karlietis, Richard C. Oude Voshaar, Jan Spijker, René J. F. Melis, Marcel G. M. Olde Rikkert

**Affiliations:** ^1^ Department of Geriatric Medicine/Radboudumc Alzheimer Centre, Donders Institute for Medical Neurosciences Radboud University Medical Centre Nijmegen The Netherlands; ^2^ Department of Geriatric Psychiatry Pro Persona Mental Health Care Nijmegen The Netherlands; ^3^ Department of Geriatric Medicine Slingeland Ziekenhuis Doetinchem The Netherlands; ^4^ Department of Psychiatry University of Groningen and University Medical Center Groningen Groningen The Netherlands; ^5^ Depression Expertise Centre Pro Persona Mental Health Care Nijmegen The Netherlands; ^6^ Behavioral Science Institute Radboud University Nijmegen The Netherlands

**Keywords:** frailty, index, functional status, geriatric psychiatry, hand grip strength, mortality, multimorbidity, nutritional status, severe psychiatric disorders, walking speed

## Abstract

**Objectives:**

Older adults with psychiatric disorders have a substantially lower life expectancy than age‐matched controls. Knowledge of risk factors may lead to targeting treatment and interventions to reduce this gap in life expectancy. In this study, we investigated whether frailty independently predicts mortality in older patients following an acute admission to a geriatric psychiatry hospital.

**Methods:**

Clinical cohort study with a 5‐year follow‐up of 120 older patients admitted to a psychiatric hospital between February 2009 and September 2010. On admission, we assessed frailty with a frailty index (FI). We applied Cox regression analyses with time to death as the dependent variable, to examine whether the FI was a predictor for mortality, adjusted for age, sex, level of education, multimorbidity (Cumulative Illness Rating Scale for Geriatrics, CIRS‐G scores), functional status (Barthel Index), neuropsychiatric symptoms (NPS), and severity of psychiatric symptoms at admission (Clinical Global Impressions Scale of Severity).

**Results:**

Of the 120 patients, 63 (53%) patients were frail (FI ≥ 0.25), and 59 (49%) had died within 5 years. The FI predicted mortality with a hazard ratio (HR) of 1.78 (95% CI, 1.06‐2.98) per 0.1 point increase, independent of the covariates. Co‐morbidity measured by the CIRS‐G and functional status measured by the Barthel Index were not significantly associated.

**Conclusions:**

Frailty was a strong predictor of mortality, independent of age, gender, multimorbidity, and functional status. This implies that frailty may be helpful in targeting inpatient psychiatric treatment and aftercare according to patients' life expectancy.

**Key points**

- Older adults with psychiatric disorders have a substantially lower life expectancy than age‐matched controls. Knowledge of risk factors may lead to targeting treatment and interventions to reduce this gap in life expectancy.
- Frailty, measured with a frailty index, is a strong predictor of mortality, independent of age, gender, multimorbidity, and functional status.
- Frailty may be helpful in targeting inpatient psychiatric treatment and aftercare according to patients' life expectancy.

## 1 INTRODUCTION

It is well recognized that adults with severe psychiatric disorders have on average a life expectancy of about 10 years less and a mortality rate two times higher than age‐matched controls.^1^, ^2^, ^3^ The excess mortality is mostly due to somatic co‐morbidity: especially cardiovascular and infectious, endocrine, pulmonary, and oncologic diseases.^1^, ^2^, ^4^, ^5^

A substantial loss in life expectancy persists in older adults with psychiatric disorders,^6^, ^7^ but risk factors may be different from those at younger age. Insight into risk factors may lead to specific interventions to reduce the gap in life expectancy between older adults with psychiatric disorders and the general population.

First, multimorbidity (having two or more chronic diseases), which includes mental disorders,^8^, ^9^, ^10^ rather than one specific disease, may be a predictor of mortality in older psychiatric patients. It is highly prevalent in older age,^11^ with a prevalence of 55% to 98% in persons 65 years or older,^12^ and is strongly associated with mortality.^13^ Secondly, functional impairment is known to be a risk factor for mortality in older general and in hospital populations and may also be a risk factor in older psychiatric patients.^14^, ^15^

Possibly most important, frailty may be a strong predictor of mortality in older patients with psychiatric disorders. This state of increased vulnerability to poor resolution of homeostasis after a stressor is known to increase the risk of adverse outcomes such as mortality,^16^, ^17^, ^18^ independently of multimorbidity and functional status in community‐dwelling older populations^17^, ^19^ as well as in older adults admitted to general or academic hospitals.^20^, ^21^ Nonetheless, to our knowledge, this has not yet been studied in depth in geriatric psychiatric patients.

The concept of frailty can be operationalized in many ways of which two models of biomedical frailty are best validated and most widely used. The first model is the accumulation of deficits model, which uses a set of symptoms, signs, disabilities, and diseases to obtain a Frailty Index (FI). A higher FI implies that a person is more frail.^22^ One item increase in 40‐item FIs resulted in 4% increase in 5‐year mortality in seven studies in community‐dwelling older populations.^23^

The second model is the “physical phenotype” model. It consists of five items: slow gait speed, weak hand grip strength, unintentional weight loss, self‐reported exhaustion, and low energy expenditure.^17^ Of these items, the last two overlap with symptoms in different psychiatric disorders. Walking speed,^24^ hand grip strength,^25^ and nutritional status^26^ have previously been investigated as sole indicators of frailty and were found to be significant predictors of mortality in older community‐dwelling populations: 0.1 m per second reduction in walking speed was associated with a 12% increase in 5‐year mortality^26^; 5‐kg reduction in hand grip strength was associated with a 16% higher 4‐year mortality.^27^ Undernutrition was predictive for 5‐ and 10‐year mortality in older adults in the community^28^, ^29^ and in hospitalized populations.^28^, ^30^

We already reported that a higher FI, lower walking speed, and multimorbidity were found to be predictors of discharge destinations with lower autonomy in patients admitted to acute wards for geriatric psychiatry.^31^ In this follow‐up study, we investigated mortality within 5 years after admission in the same study population. We primarily focused on the question whether frailty, measured with a FI, is a predictor of mortality, independent of age, sex, level of education, multimorbidity, functional status, severity of the psychiatric symptoms at admission, and a diagnosis of cognitive disorders with neuropsychiatric symptoms (NPS). Secondly, we investigated three other frailty measures as possible predictors for mortality: walking speed, hand grip strength, and nutritional status.

## 2 METHODS

We conducted a 5‐year follow‐up study in a prospectively sampled clinical cohort of 120 older adults, admitted to acute wards for geriatric psychiatry between 1 February 2009 and 1 August 2010. The methods have been described previously and are summarized here.^31^

### 2.1 Setting and participants

The study was carried out in two acute geriatric psychiatry wards of Pro Persona Mental Health Care, a large psychiatric teaching hospital in Nijmegen, the Netherlands. Eligible were all consecutively referred patients. Excluded were patients who declined informed consent, were admitted less than 5 days, or were not able to understand Dutch. If patients were judged incapable to consent themselves, we asked their proxies. As we conducted an observational study with only limited extra data collection compared with our usual care, the medical ethical committee approved informed consent as “written or oral consent of the patient or proxy.” In patients who were readmitted (n = 30), only the data of the first included admission were analysed in our study. The 172 consecutive admissions pertained to 142 unique patients in the study period. As 10 patients refused consent and 12 were excluded according to our exclusion criteria, the final study sample consisted of 120 patients.^31^

### 2.2 Demographics and psychiatric diagnoses

On admission, we collected data on age, sex, marital status, level of education, and living situation. All patients were clinically diagnosed according to the DSM‐IV‐TR classification.^32^ We used the main diagnosis for our study. We categorized all patients in four main diagnosis groups: depressive disorder (n = 41); cognitive disorder and dementia, admitted with NPS (n = 41); psychosis and bipolar disorder (n = 17), and other psychiatric diagnoses (n = 21; anxiety disorder: n = 5, somatoform disorder: n = 4, substance abuse disorder: n = 5, adjustment disorder: n = 5, and personality disorder: n = 2).

### 2.3 Frailty

We constructed an FI of 39 items^31^ following the procedure described by Searle et al (Data S1).^33^ The FI ranges between 0 and 1, as the sum score of the deficits that are present is divided by the number of deficits that can be scored. FI scores smaller than 0.08 indicate being robust, a score between 0.08 and 0.25 indicates a prefrailty state, and a score greater than 0.25 indicates being frail.^34^, ^35^

Walking speed was measured as the average speed in meters per second over 6‐m walking.^24^ Generally, a walking speed of >1.0 m/s is judged as good, and <0.8 indicates probable frailty.^17^ Hand grip strength was measured in kilogram force (kg), with the Jamar dynamometer, using the dominant hand. Overall, a hand grip strength of >18 and >30 kg are considered to be adequate for women and men, respectively.^17^ We used the Mini Nutritional Assessment (MNA) as measure of nutritional status (range 0‐30: score < 17 indicating undernutrition, 17‐23.5: risk for undernutrition, and 24‐30: well nourished).^36^

### 2.4 Multimorbidity and functional status

Multimorbidity was measured with the Cumulative Illness Rating Scale for Geriatrics (CIRS‐G).^37^ It measures the cumulative burden of diagnosed diseases in 14 domains: 13 domains of different somatic organ systems and the psychiatric domain. Each item can be scored from 0 to 4 (range 0‐56, higher score: more multimorbidity). Functional status was measured by assessing the performance on activities of daily living with the Barthel Index (range 0‐20, higher score: more independent).^38^

### 2.5 Severity of the psychiatric disorder

To assess the severity of the mental disorders, we used the Clinical Global Impressions Scale of Severity at Admission (CGI‐SA).^39^ The CGI‐SA provides an overall clinician‐determined summary measure that takes into account all available information, including knowledge of the patient's history, psychosocial circumstances, symptoms, behaviour, and the impact of the symptoms on the patient's ability to function.^40^ The CGI‐SA is a 7‐point scale scoring from 1 (normal, no symptoms) to 7 (very severely ill). We asked an expert panel of three professionals, who were not involved in the treatment of the included patients, to independently score the CGI‐SA for each patient retrospectively. The ICC for the CGI‐SA was good with a score of 0.77.^31^

### 2.6 Data collection

All measurements, including the items incorporated in the FI, were conducted by professionals who were involved in patient care of the participants: two residents in training for geriatrician and one in training for psychiatrist, under supervision of one psychiatrist and two geriatricians. Nurses scored the Barthel Index, a physiotherapist the mobility measures, and a dietician the body mass index (BMI) and MNA.

### 2.7 Outcome measure: mortality

We analysed survival over 1 and 5 years after admission. Mortality was checked in the national mortality registry of the Netherlands for date of death until 5 years after the last discharges from the acute wards.

### 2.8 Analysis

Univariable associations with survival times were graphically assessed with Kaplan Meier curves and tested with a logrank test. Multicollinearity was checked using the Variance Inflation Factor (VIF).

We used Cox proportional hazards regression models to analyse the predictors' association with survival times, first in a univariable and next in a multivariable manner.

As predictors of survival in our primary analyses, we considered age, sex, level of education (low vs middle/high), diagnosis (patients with NPS vs patients with other diagnoses), CGI‐SA, FI, CIRS‐G, and the Barthel Index.

In our secondary multivariable models, we considered age, sex, level of education, NPS, and CGI‐SA, combined with either walking speed, hand grip strength, or the MNA score. Statistical analyses were conducted using SPSS version 25, with a significance level of .05.

## 3 RESULTS

### 3.1 Baseline characteristics

Demographics, psychiatric, and geriatric measures of the 120 participants at admission are presented in Table 1. Mean age of the whole sample was 74.6 (SD: 7.8) years, and 62% were female. A total of 63 (53%) patients had an FI ≥ 0.25, 55 (49%) had a walking speed < 0.8 m/s or were unable to walk, 52 (47%) patients had a low hand grip strength, 39 (34%) were undernourished, and 66 (58%) patients were at risk for undernutrition.

**Table 1:** Baseline characteristics for the whole sample and for patients who survived or deceased over five years after admission

	Total sample N=120	Survivor after 5 years N=61	Deceased within 5 years N=59
**Age**	74.6 (7.8)	71.3 (7.2)	78.0 (6.9)
**Sex**			
women	74 (62)	45 (74)	29 (49)
men	46 (38)	16 (26)	30 (51)
**Marital status**			
Married or with spouse	49 (41)	28 (46)	21 (36)
Never married or widowed	71 (59)	33 (54)	38 (64)
**Level of Education** (n =110)			
Middle and High	48 (44)	29 (51)	19 (36)
Low	62 (56)	28 (49)	34 (64)
**Living situation**			
Independent (alone or with spouse)	94 (78)	51 (84)	43 (73)
Not independent	26 (22)	10 (16)	16 (27)
Nursing home	10 (8)	3 (5)	7 (12)
Residential home	13 (11)	5 (8)	8 (14)
Sheltered Care Mental Care	3 (3)	2 (3)	1 (2)
**Diagnosis DSM IV**			
Depressive disorders	41 (34)	22 (36)	19 (32)
Cognitive disorders (NPS)	41 (34)	13 (21)	28 (48)
Psychosis and Bipolar disorders	17 (14)	11 (18)	6 (10)
Other diagnoses	21 (18)	15 (25)	6 (10)
**Frailty Index**	0.27 (0.10)	0.23 (1.0)	0.31 (0.10)
< 0.08	5 (4)	5 (8)	0 (0)
0.08 ≤ FI < 0.25	52 (43)	33 (54)	19 (32)
0.25 ≤ FI ≤ 0.45	56 (47)	22 (36)	34 (58)
> 0.45	7 (6)	1 (2)	6 (10)
**Walking speed 6 meter** (m/sec) (n = 112)	0.85 (0.33)	0.92 (0.36)	0.76 (0.28)
> 1.0	31 (28)	21 (35)	10 (19)
0.8 – 1.0	21 (19)	14 (23)	7 (14)
< 0.8	50 (45)	19 (32)	31 (60)
Mobility too impaired to test	5 (5)	3 (5)	2 (4)
Not able for other reasons	5 (5)	3 (5)	2 (4)
**Hand Grip Strength** (n = 112)			
Women (n = 72)	19.6 (7.7)	21.5 (6.9)	16.4 (8.0)
Men (n = 40)	30.7 (8.7)	37.3 (8.4)	26.8 (6.4)
**Nutritional status** (n = 114)			
Mini Nutritional Assessment (0 ‐30)	18.1 (4.4)	18.4 (4.4)	17.8 (4.6)
24 – 30	9 (8)	6 (10)	3 (5)
17 – 23.5	66 (58)	32 (55)	34 (61)
< 17	39 (34)	20 (35)	19 (34)
**Multimorbidity**			
Cumulative Index Rating Scale Geriatrics (0‐56)	13.5 (5.4)	11.7 (5.0)	15.4 (5.1)
**Functional status** (n = 116)			
ADL Barthel index (0 ‐ 20)	15.4 (5.3)	16.8 (4.9)	14.0 (5.3)
ADL Barthel 19 – 20	52 (45)	35 (59)	17 (30)
ADL Barthel 1 – 18	64 (55)	24 (41)	40 (70)
**Cognition** (n = 114)			
MMSE (0‐30)	22.9 (6.5)	23.9 (6.1)	21.7 (6.7)
**CGI‐SA**	5.2 (0.76)	5.1 (0.73)	5,3 (0.77)

*Note*: Continuous variables: mean and standard deviation (SD); categorical variables: N = number and (%). NPS: NeuroPsychiatric Symptoms; MMSE: Mini Mental Status Examination; N=120 unless stated otherwise.

### 3.2 Mortality

One year after admission, 20 (16%) of patients had died. All had a FI ≥ 0.25. All had a FI ≥ 0.25.

Five years after admission, 59 patients (49%) had died: 65% of the men and 39% of the women. There was a significant higher mortality among men compared with women (*P* logrank = .002), with a median survival time of 2.9 years for men.

**Figure 1:**
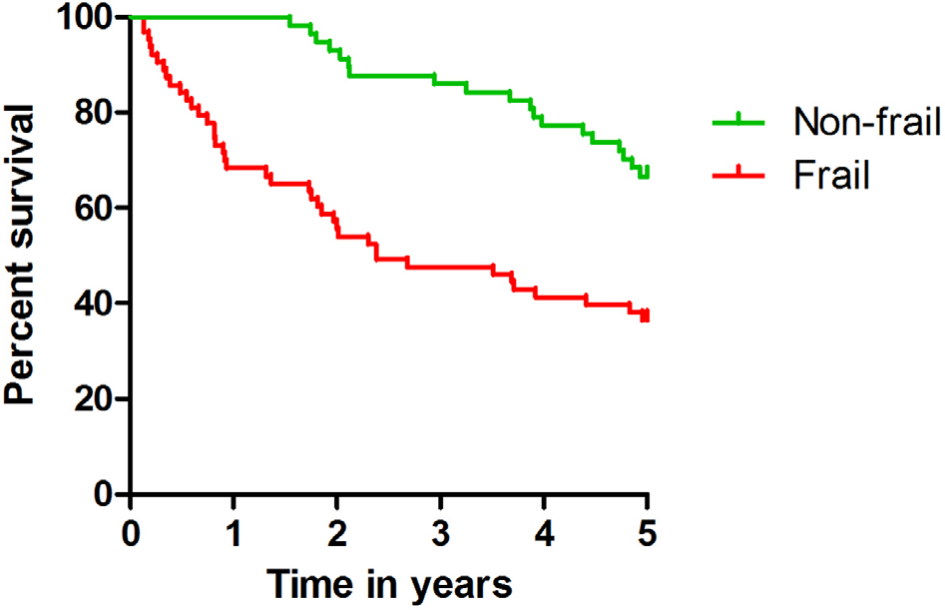
FI ≤ 0.25 versus >0.25 and 5‐year survival

The mortality rate of patients with an FI > 0.25 was 63% with a median survival time of 2.4 years, whereas 33% of patients with an FI ≤ 0.25 died within the first 5 years after admission (*P* logrank < .001); see Figure 1.

We found no significant multicollinearity between the variables, as the VIF was between 1.00 and 2.43.

### 3.3 FI and mortality within 1 year after admission

We analysed the predictive value of the FI per 0.1 point increase in multivariable analysis with only age and sex as covariates. The hazard ratio (HR) was 2.73 (95% CI, 1.80‐4.15) per 0.1 point increase in which age lost its predictive value. The group of patients that died within 1 year was too small for a complete multivariable analysis.

### 3.4 FI, CIRS‐G, Barthel, and mortality within 5 years after admission

The FI, CIRS‐G, and the Barthel Index were each predictive for mortality within 5 years after admission in both the univariable Cox regression analyses and in models adjusted for age and sex (see Table 2).

**Table 2:** Results of Cox proportional hazards regression analysis for mortality during 5‐year follow‐up

	Univariable			Age‐ and Sex‐Adjusted Model^a^			Fully Adjusted Model (n = 110)		
Variable and coding	HR	95% CI	*P* value	HR	95% CI	*P* value	HR	95% CI	*P* value
Age (y)	1.10	1.06‐1.14	<.01				1.10	1.05‐1.16	.00
Sex (men vs women)	2.24	1.34‐3.74	<.01				2.29	1.17‐4.45	.02
Education (low vs middle and high)	1.66	0.95‐2.91	.08	1.84	1.01‐3.33	.05	0.96	0.50‐1.85	.91
FI (per 0.1)	1.87	1.48‐2.36	<.01	1.81	1.41‐2.31	<.01	1.78	1.06‐2.98	.03
Barthel (per point)	0.92	0.88‐0.96	<.01	0.94	0.89‐0.98	<.01	1.06	0.98‐1.13	.14
CIRS‐G (per point)	1.10	1.05‐1.15	<.01	1.09	1.04‐1.14	<.01	1.06	0.98‐1.15	.16
Diagnosis (NPS vs other diagnoses)	2.33	1.39‐3.89	<.01	1.87	1.11‐3.13	.02	1.98	1.00‐3.95	.05
CGI‐SA (per point)	1.49	1.02‐2.17	.04	1.38	0.95‐1.99	.09	1.51	0.99‐2.30	.06

Abbreviations: CGI‐SA: Clinical Global Impressions Scale of Severity on admission; CIRS‐G: Cumulative Illness Rating Scale Geriatrics; FI, frailty index; NPS, neuropsychiatric symptoms.

a Results from partially adjusted models in which covariates were included separately, adjusted only for age and sex.

In our fully adjusted model including NPS versus other diagnoses and severity of psychiatric disorder (CGI‐SA), and entering the FI, CIRS‐G, and Barthel simultaneously, the FI (HR 1.78 [95% CI, 1.06‐2.98] per 0.1 point increase) remained predictive for mortality besides age and sex but not the CIRS‐G or Barthel Index.

### 3.5 Walking speed, hand grip strength, MNA scores, and mortality within 5 years after admission

In our secondary analyses, we found walking speed and hand grip strength to have predictive value for mortality within 5 years after admission when adjusted for age and sex. For lower walking speed, we found a HR 1.11 (95% CI, 1.01‐1.21) per 0.1 m per second, and for hand grip strength, a HR of 1.43 (95% CI, 1.06‐1.92) per 1 kg less strength. Nutritional status was not significantly associated with mortality, with a HR of 0.97 (95% CI, 0.91‐1.03) per point increase in MNA score. In the fully adjusted models, neither walking speed (HR 1.06 [95% CI, 0.96‐1.17) nor hand grip strength (HR 1.28 [95% CI, 0.91‐1.80] remained predictive for mortality.

## 4 DISCUSSION

In this first study examining the predictive value of frailty on mortality of older adults admitted to geriatric psychiatric wards, the 5‐year mortality rate was 49%. After adjusting for age, sex, level of education, severity of psychiatric disorder, and a diagnosis of NPS, frailty remained a significant predictor of mortality independent of multimorbidity and functional status.

### 4.1 Survival time

The survival time of our population indicates a sharp reduction in life expectancy compared with the general population, especially for men. The mean life expectancy of men aged 75 years in 2010 was 10.8 years in the general Dutch population with a mean 5‐year survival rate of 79% for men and 87% for women. This is substantially higher than the 35% survival in men and 61% in women we found.^41^

Two population‐based studies on persons aged 65 years and over with severe psychiatric disorders showed a decreased life expectancy of 3 years in men compared with age‐matched controls across a follow‐up period of 12 to 14 years.^6^ Another study found a 10‐year mortality rate of 66% for men and 56% for women with schizophrenia.^7^ The high level of frailty in our inpatient population, compared with older persons in population‐based cohort studies, may explain the higher mortality rate in our population compared with these studies.

### 4.2 FI and mortality

A systematic review found that the effect of frailty on mortality may persist for 5 years in community‐dwelling older populations.^21^ Our study confirms this long‐term predictive value of frailty on mortality and underlines the excess loss of life expectancy in frail patients in our older psychiatric population.

Only one study in a recent systematic review reported an HR of a FI per 0.1 point increase, as we did, and found an HR of 1.25 (95% CI, 1.20‐1.30) on 5‐year mortality, corrected for age and sex, in a community‐dwelling population, where we found an HR of 1.81.^42^ The high HR of 1.78 in our fully adjusted model may be explained by the higher mean FI in our clinical population. Anyhow, we confirm the association of frailty with mortality in the older psychiatric population.

Former studies have found frailty, functional impairment, and multimorbidity to be three distinct but associated concepts.^17^, ^42^, ^43^ Frailty was found to be a predictor of mortality independent of multimorbidity and functional impairment in community‐dwelling population‐based studies.^17^, ^19^, ^42^ Our results are in line with these findings.

The inclusion of the presence of somatic disease items in our FI probably explains why multimorbidity loses predictive value in our full model. This is supported by the aforementioned study, which showed that when multimorbidity and functional status items were excluded from the FI, both disability and multimorbidity contributed to prediction of mortality, but when these items were included in the FI, disability and multimorbidity were no longer predictive.^42^

We found that functional impairment was not independently predictive for mortality. This might partly be explained by the fact that functional status is also included in the FI, although to a lower extent than multimorbidity. A second and probably important explanation is the fact that functional impairment can be caused by psychiatric disorders and may improve when these disorders recuperate, hence losing their predictive power on the long term. This is in line with the results of our former study showing that functional status at admission was not predictive of outcomes at discharge.^31^

The comparison of our findings with studies with in‐hospital patients is not merely hampered by differences in follow‐up duration (1 year at most) and by differences in the operationalization of the FI and our small sample size.^44^, ^45^, ^46^, ^47^ One study on patients admitted to geriatric wards found an HR of 1.91 (95% CI, 1.6‐2.3) per 0.1 FI‐point increase for mortality (corrected for age and sex), with a follow‐up period of 1 year and an overall mortality rate of 20%. The mortality rate of 16% and the HR for the FI per 0.1 point (corrected for age and sex) of 2.73 (95% CI, 1.80‐4.15) that we found for 1‐year mortality is at least comparable with these outcomes.

### 4.3 Walking speed, hand grip strength, undernutrition and mortality

Half of the patients showed a low walking speed and hand grip strength, but our study could not confirm the predictive value of these factors for mortality found in meta‐analyses among community‐dwelling older persons.^24^, ^26^, ^27^, ^48^, ^49^ Our findings indicate that walking speed and hand grip strength as sole measures of frailty do not have a similar predictive value compared with an FI in a psychiatric sample. There are several possible explanations for the lack of predictive value. Particularly in a psychiatric sample, walking speed and hand grip strength might become less reliable because of a temporarily lowered level of motivation. Probably, because of the relatively small sample size, there is a lack of power, as the trend of the HRs for both walking speed and hand grip was in the direction that was expected in our multivariable analysis but did not reach significance. The MNA was not predictive for mortality at all in our population, probably explained by the fact that the effect of malnutrition is (sub)acute and related to the psychiatric disorders. Patients often improve their nutritional status when recuperating and for that reason MNA may not be predictive in the long term in this population.

### 4.4 Strengths and limitations

Our study has strong points: It is the first to take frailty, multimorbidity, and functional status into account as predictors for mortality in older patients with severe psychiatric disorders. Moreover, it presents a well‐described clinical study population and has complete follow‐up data. However, the small sample size is a limitation. It hampers the possibility of analysing differences between all four diagnosis groups and probably limits the power to detect a predictive effect of not only walking speed or hand grip strength but also of the psychiatric characteristics (eg, the severity of the psychiatric disorder [CGI‐SA] or a diagnosis of NPS). As we only studied patients in one psychiatric hospital, we realize that our patients may not be representative for other departments of geriatric psychiatry. However, the observed association between frailty and mortality is likely generalizable to other settings, as this association is consistent with previous studies in other populations.

### 4.5 Practice implications and further research

We found a high level of frailty when patients are admitted to acute geriatric psychiatric wards and a high impact of frailty on mortality, extending over 5 years.

This implies that frailty may be helpful in targeting patient psychiatric treatment and aftercare according to patients' life expectancy. It is used as such in general hospitals, for instance, to support targeted end‐stage renal dysfunction treatment^50^ or interventions such as aortic valve replacement.^51^

To realize more widespread use of frailty measures, an FI might be incorporated in digital medical records.^52^ Another option is using simpler frailty screening instruments, such as the clinical frailty scale, which is validated against the FI,^53^ combined with a multimorbidity measure.

Our data support the added value of frailty assessment in geriatric psychiatry populations, which in analogy to general medical populations may be used to identify patients who can benefit from a comprehensive geriatric assessment (CGA).^54^ A CGA can result in specific advices on prevention and treatment and thus may also help to reduce the high mortality figures present in these frail older psychiatric patients.

Frailty should be the focus of further research to improve outcomes in older psychiatric patients. Future studies should examine the effectiveness, efficiency, and feasibility of using frailty‐based screening methods in clinical practice to improve treatment‐related decision making and the effect of possible interventions on outcomes in older psychiatric patients.

## 5 CONCLUSION

Frailty is a strong predictor of mortality in older adults, who are acutely admitted to geriatric psychiatric wards, independent of age, gender, multimorbidity, and functional status. This implies that frailty may be helpful in targeting inpatient psychiatric treatment and aftercare according to patients' life expectancy. Frailty should be the focus of further research to improve outcomes in older psychiatric patients.

## CONFLICT OF INTEREST

None declared.

## Supporting information

Data S1: Operationalisation of 39‐item Frailty Index (separate file)

## Data Availability

The data that support the findings of this study are available from the corresponding author upon specified request.
