# Supplementary material for: Frailty as a predictor of mortality in older adults within 5 years of psychiatric admission
Source: Int J Geriatr Psychiatry. 2020 Feb 23;35(6):617–25. doi: 10.1002/gps.5278 (PMC7317407; doi:10.1002/gps.5278)
Supplement: Supplementary file 1 — Data S1: Operationalisation of 39‐item Frailty Index (separate file) [file GPS-35-617-s001.docx]

**E-table 1: Operationalisation of 39-item Frailty Index**

| **Items** | **Scores** |
| --- | --- |
| **Functional status** |  |
| Transfer | 0 = independent; 0.5 = some help ; 1 = much help or dependent |
| Global Impression Mobility | 0 = independent; 0.5 = walking aid 1 = wheelchair or bedridden |
| IADL | 0 = Independent; 0.5 = some help; 1 = much help or dependent |
| Impaired vision | 0 = No; 1 = Yes |
| Impaired hearing | 0 = No; 1 = Yes |
| **Cognition** |  |
| MMSE (0 – 30) | 0 = 25 – 30; 0.25 = 21 – 24; 0.50 = 18 – 20; 0.75 = 10 – 17 ; 1 = 10 |
| Difficulty in practical skills | 0 = No; 1 = Yes |
| Difficulty in understanding | 0 = No; 1 = Yes |
| Difficulty in speaking | 0 = No ; 1 = Yes |
| **Medical history according to own general practitioner** |  |
| Hypertension | 0 = No; 1 = Yes |
| Acute coronary syndrome | 0 = No; 1 = Yes |
| Congestive heart failure | 0 = No; 1 = Yes |
| Chronic Pulmonary Disease | 0 = No; 1 = Yes |
| Cancer | 0 = No; 1 = Yes |
| Stroke | 0 = No; 1 = Yes |
| Parkinsonism | 0 = No; 1 = Yes |
| Thyroid disease | 0 = No; 1 = Yes |
| Diabetes Mellitus | 0 = No; 1 = Yes |
| Arthritis/osteoporosis | 0 = No; 1 = Yes |
| Kidney disease | 0 = No; 1 = Yes |
| **Physical examination** |  |
| Tremor | 0 = No; 1 = Yes |
| Impression of illness; deconditioning; undernutrition | 0 = No; 1 = Yes |
| Abnormal auscultation lungs | 0 = No; 1 = Yes |
| Abnormal auscultation heart | 0 = No; 1 = Yes |
| RR systolic | 0 = 101 – 160; 1 = < 101 or >160 |
| **Lab hematology and chemistry** |  |
| Hemoglobin (mmol/l) | Female = 0 = 7.5 – 10.0; 0.5 = 7;0 – 7;5; 1 = < 7.0 OR > 10.0  Male = 0 = 8.5 – 11.0; 0.5 = 8.0 – 8.5; 1 = < 8.5 OR > 11.0 |
| CRP (mg/l) | 0 = ≤ 3; 0.5 = 3 – 10; 1 = > 10 |
| MDRD (ml/min) | 0 = > 60; 0.5 = 50 – 60; 1 = < 50 |
| Glucose (mmol/l) | Fasting 0 = 4.0 – 6.1; 0.5 = > 6.1 OR ≤ 7.1; 1 = > 7.1  Non fasting 0 = 4.0 – 7.8; 0.5 = > 7;8 OR ≤ 11.1; 1 = < 4.0 OR > 11.1 |
| Natrium (mmol/l) | 0 = 135 – 145 ; 0.5 = 130 – 134 OR 146 – 150; 1 = < 130 OR > 150 |
| Kalium (mmol/l) | 0 = 3.5 – 5.0; 0.5 = 3.0 – 3.4 OR 5.1 – 5.5; 1 = < 3.0 OR > 5.5 |
| TSH (mU/l) | 0 = 0.27 – 4.2; 0.5 = 4.3 – 10.0; 1 = < 0.27 OR > 10.0 |
| **Number of medications at admission ≥ 4** | 0 = ≤ 4; 1 = > 4 |
| **Nutrition** |  |
| BMI (kg/m^2^) | 0 = 23 ≥ x ≤ 33; 0.5 = 19 – 23 OR > 33 ; 1 = < 19 OR > 35 |
| Involuntary weight loss last three months (kg) | 0 = No weight loss; 0.25 = don’t know; 0.5 = 1- 3; 1 = ≥ 3 |
| **Mobility and muscle strength** |  |
| Fall last half year | 0 = No; 1 = Yes |
| TUGT (sec) | 0 = < 10; 0.5 = 10 – 20 ; 1 = > 20 |
| POMA (0 - 28) | 0 = > 24; 0.5 = 19-24; 1 = < 19 |
| Fear of falling | 0 = No; 1 = Yes |

MMSE: Mini Mental Status Examination (Folstein 1975) TUGT: Timed Up and Go Test (Podsiaslo 1991); POMA: Performance Oriented Mobility Assessment (Tinetti 1986)
